# Validation of a Dutch version of the Actionable 8-item screening questionnaire for neurogenic bladder overactivity in multiple sclerosis: an observational web-based study

**DOI:** 10.1186/s12955-015-0368-4

**Published:** 2015-10-30

**Authors:** Peter Joseph Jongen, Bertil F. M. Blok, John P. Heesakkers, Marco Heerings, Wim A. Lemmens, Rogier Donders

**Affiliations:** Department of Community and Occupational Medicine, University Medical Centre Groningen, University Groningen, Antonius Deusinglaan 1, 9713 AV, Groningen, The Netherlands; MS4 Research Institute, Ubbergseweg 34, 6522 KJ Nijmegen, The Netherlands; Department of Urology, Erasmus University Medical Center, P.O. Box 2040, 3000 CA Rotterdam, The Netherlands; Department of Urology, Radboud University Nijmegen Medical Center, P.O. Box 9101, 6500 HB Nijmegen, The Netherlands; MH-Advies and Organisatiebureau, IJselstraat 81, 9406 TR Assen, The Netherlands; Department for Health Evidence, Radboud University Nijmegen Medical Center, P.O. Box 9101, 6500 HB Nijmegen, The Netherlands

## Abstract

**Background:**

In patients with multiple sclerosis (MS) the impact of urological symptoms on quality of life and daily activities is considerable. Yet, a substantial percentage of patients may not be urologically evaluated and thus fail to be treated concordantly. The 8-item Actionable questionnaire is a validated English screening tool for the detection of neurogenic bladder overactivity in MS. To enable the use of the 8-item Actionable in The Netherlands and Belgium we translated the questionnaire into the Dutch language and investigated the test-retest reliability and the concurrent validity of the Dutch version.

**Methods:**

The process of translating the English Actionable questionnaire into the Dutch language included forward translations and back-translations. Then, in an online observational study, MS patients completed the Dutch Actionable at Days 1 and 8, and the Multiple Sclerosis Quality of Life 54-Items (MSQoL-54) and Multiple Sclerosis Impact Profile (MSIP) questionnaires at Day 1; the Expanded Disability Status Scale (EDSS) score was assessed by phone at Day 1. For assessment of the test-retest reliability Pearson’s correlation coefficient (r) between the Day 1 and Day 8 Actionable scores was calculated. For assessment of the concurrent validity r values were calculated between the Day 1 Actionable score and the EDSS score, the Physical and Mental MSQoL-54 composites, and the MSIP domain and symptom disability scores.

**Results:**

Study population: *N* = 141 (106 female, 35 male) (80 relapsing remitting, 48 progressive, 13 unknown), mean age 47.8 (standard deviation [SD] 10.4) years, mean EDSS score 4.7 (SD 1.8); 137 patients completed the Day 8 assessment. Pearson’s r between Actionable scores Day 1 and Day 8: 0.85 (*P* < .0001). Pearson’s r between Actionable score Day 1 and scores for EDSS 0.41 (*P* < 0.0001), MSQoL-54 Physical −0.31 (*P* = 0.0002), MSQoL-54 Mental −0.29 (*P* = 0.0005), MSIP Excretion and Reproductive Functions 0.44 (*P* < 0.0001), Muscle and Movement Functions 0.39 (*P* < .0001), Basic Movement Activities 0.37 (*P* < 0.0001), Activities of Daily Living 0.32 (*P* < 0.0001), Participation in Life Situations 0.29 (*P* = 0.0006) and Mental Functions 0.20 (*P* = 0.0189).

**Conclusions:**

The Dutch version of the Actionable urological screening tool for MS shows a good test-retest reliability and a good concurrent validity with disabilities and HRQoL.

## Background

Multiple sclerosis (MS) is a chronic inflammatory and degenerative disease of the central nervous system, mostly affecting young adults. Up to 80 % of MS patients will suffer from some degree of bladder dysfunction in the course of the disease, the most prominent and disabling symptoms being urinary incontinence, urge and frequency of urination [1]. The impact of MS-related urological symptoms on health-related quality of life (HRQoL) and daily activities is considerable [2]. Moreover, a recent international study showed that in patients with neurogenic overactive bladder the occurrence of incontinence was associated with significantly higher health care resource utilization, lower HRQoL and lower productivity [3].

Notwithstanding the clinical and societal consequences of overactive bladder symptoms in MS, there is evidence that in clinical practice a substantial percentage of patients is not being adequately diagnosed and treated [4]. A large scale survey of the North American Research Committee On Multiple Sclerosis (NARCOMS) patient registry identified at least one moderate to severe urinary symptom in 65 % of the respondents, and showed that of patients with moderate to severe overactive bladder symptoms only 43 % were evaluated by urology, that 51 % were treated with anticholinergic medication, and that newer treatments were significantly underused (less than 10 % total use) [5]. These findings indicate that despite an increasing awareness of overactive bladder symptoms and the need for evaluation and treatment, many MS patients’ symptoms still remain unnoticed and underserved [5, 6].

The 16-items Actionable Bladder Symptom Screening Tool (ABSST) was developed in response to the growing need for an accurate way to identify MS patients with bladder problems [4]. This psychometrically validated questionnaire identifies MS patients with bladder symptoms who might benefit from neurogenic detrusor overactivity-specific treatment [4]. The ABSST asks questions to uncover whether patients are experiencing urinary symptoms, to what degree, what the effects of these symptoms are on daily life, and whether the bladder issues warrant a visit to a urologist [4]. Soon, a short form of the 16-items ABSST, the 8-items Actionable questionnaire was developed, which demonstrated similar psychometric properties and equally high sensitivity and specificity as the longer form tool [7].

Health care professionals may consider using the Actionable questionnaire as a routine standard of care in their daily practice. It may help to identify those MS patients who are likely to benefit from an urologic referral for distressing bladder symptoms. The use of the Actionable by patients for self-screening purposes in the context of self-management is also conceivable. To enable the use of the Actionable in The Netherlands and Belgium, we translated the original English questionnaire into Dutch and evaluated the test-retest reliability and concurrent validity of this Dutch version. We demonstrated the Dutch Actionable to have a good test-retest reliability (Pearson’s *r* = 0.85; *P* < 0.0001) and a good concurrent validity with the Expanded Disability Status Scale (EDSS) score (*r* = 0.41; *P* < 0.0001), and physical (*r* = −0.31; *P* = 0.0002) and mental (*r* = −0.29; *P* = 0.0005) HRQoL.

## Methods

### Study design and setting

The Dutch Actionable validation study was an observational, non-interventional, web-based study in The Netherlands in the period January to May 2015. The study was investigator-initiated and investigator-sponsored with financial support from Allergan Pharmaceutical Ireland Inc.

Inclusion criteria for participation were 1) having been diagnosed with MS, 2) no relapse in the last 30 days, and 3) willing and able to comply with the requirements of the protocol, i.e. to complete online questionnaires and to have disability assessed by phone by an MS nurse (MH). Patients were informed about the possibility to participate by the urologists and continence nurses of the urological departments of the Erasmus Medical Center and the Radboud University Nijmegen Medical Center, by neurologists and MS nurses in The Netherlands, by postings on the websites of the patient organizations Nationaal Multiple Sclerose Fonds, Multiple Sclerose Vereniging Nederland and Multiple Sclerose Anders, and the website of the MS4 Research Institute. Patients participating in the web-based Dutch Multiple Sclerosis Study, a prospective long-term assessment of HRQoL and disabilities in MS, were informed via e-mail. The study information and the consent form were available as download on the website of the MS4 Research Institute www.ms4ri.nl. The information given to potential participants concerned the purpose of the study, the eligibility criteria, the kind of data that were obtained, where the data were stored, and who the principal investigator (PJJ) was. No incentives were offered.

After having completed informed consent online and confirmed their participation by sending their confirmation, the patients received a personal code and logged on to the website of the MS4 Research Institute, to choose a username and password. The study was performed using the LimeSurvey software, an open source online application. There was no testing of the MS4 Research Institute’s platform for this study since it was already being used in various research projects. The items of the questionnaire were fixed. The responses were automatically captured. To protect the personal data from unauthorized access various mechanisms were used to comply with European Union regulations concerning online medical data, including the use of a personal username and a strong password, separation in the database of personal information from the answers to the questions, each screen having a username and password protection, VPN tunnelling, 256-bits encryption, and the encryption of the participants’ identities via unique 15 digits codes. Automated completeness checks were done before questionnaires could be submitted. The respondents saw an overview of all questions and answers before submission and they could change the answers before submitting. After submission changes were no longer possible. Only completed questionnaires were analyzed. The help desk (MH) contacted respondents by phone in case they did not succeed in completing questionnaires. No methods were used to adjust for an non-representativeness of the sample. The study protocol was presented to the ethical committee Medisch Ethische Toetsingscommissie Brabant (METC) (nr NW2015-08) and the committee concluded that a review was not indicated, as the study did not qualify for being tested according to the Dutch Medical Research Involving Human Subjects Act of 1999 (www.wetten.overheid.nl/BWBR0009408) [8]. The study was performed in agreement with the Declaration of Helsinki (Ethical Principles for Medical Research Involving Human Subjects version 2013; 64th World Medical Association General Assembly, Fortaleza, Brazil, October 2013) (www.wma.net) and the Wet medisch-wetenschappelijk onderzoek met mensen (WMO) (www.wetten.overheid.nl/BWBR0009408).

The first patient was included on 24^th^ February 2015 and the last patient on 28^th^ April 2015. The closure of the database was May 7^th^ 2015.

### Actionable questionnaire

The 8-item Actionable was developed from the 16-item ABSST [4, 7] and, as in the ABSST, each item is scored from 0 (no symptoms or impact) to 3 (extreme symptoms or impact). The Actionable score is calculated as the sum of the item scores on the instrument (total score) and ranges from 0 (minimum) to 24 (maximum). Item 9 asks whether the patient would like to receive help for his or her bladder symptoms (Yes or No). This item does not contribute to the score.

### Health-Related Quality of Life

HRQoL was assessed by the Multiple Sclerosis Quality of Life 54-Item (MSQoL-54) questionnaire [9]. The MSQoL-54 is a psychometrically validated, MS-specific, multi-dimensional inventory of patient-centred health status, and consists of the Short Form 36-Item (SF-36) health survey as a generic core measure, supplemented with 18 questions on items relevant to MS patients in the areas of health distress, sexual function, satisfaction with sexual function, overall quality of life, cognitive function, energy, pain and social function [9]. The MSQoL-54 contains 52 items distributed into 12 scales, and two single items. A physical and a mental dimension underlie the MSQoL-54: the Physical and Mental composites [9]. Scores for the Physical and Mental composites range from 0 to 100, where higher values indicate better HRQoL [9].

### Disabilities

Disability was measured by use of the Expanded Disability Status Scale (EDSS) via telephone [10] and by the Multiple Sclerosis Impact Profile (MSIP) questionnaire online. The classical EDSS is based on a neurological examination that provides the basis for the assessment of several functional systems that, according to predefined algorithms, contribute to the EDSS score [11]. An EDSS version for use by telephone via a structured interview has been developed and validated [10].

The MSIP comprises 36 questions assessing disability (Q1a-Q36a) and disability perception (Q1b-Q36b) in the domains Muscle and Movement Functions (MMF), Excretion and Reproductive Functions (ERF), Basic Movement Activities (BMA), Activities of Daily Living (ADL), Participation in Life Situations (PLS), Environmental Factors (EF), Mental Functions (MF) and the symptoms Fatigue, Pain, Speech and Vision [12, 13]. The MSIP yields validated domain scores, ranging from 0 to 12 (ERF, MF), 0 to 15 (BMA), 0 to 16 (MMF), 0 to 20 (EF), 0 to 24 (ADL), and 0 to 26 (PLS), and symptoms scores, ranging from 0 to 4. Higher scores indicate a worse condition.

### Schedule of assessments

The Actionable questionnaire was completed at baseline (Day 1) and after one week (Day 8). The MSQoL-54 and the MSIP were completed at Day 1. The EDSS score was assessed at Day 1.

### Statistics

For assessment of the test-retest reliability Pearson’s coefficient was calculated for the correlation between the Day 1 and Day 8 Actionable scores. For assessment of the concurrent validity Pearson’s correlation coefficients were calculated between the Day 1 Actionable score and the Physical and Mental MSQoL-54 scores, the EDSS score, and the MSIP domain and symptom disability scores. P values lower that 0.05 were considered statistically significant. For the test-retest reliability to be qualified as good an r value higher than 0.70 was required.

## Results

### Development of Dutch version of actionable

**Fig. 1:**
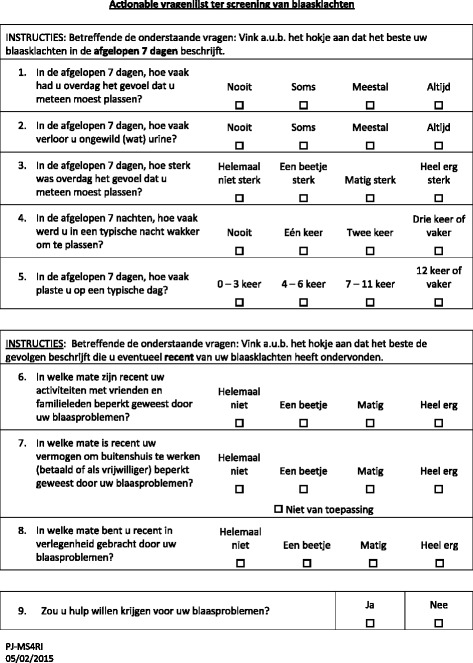
Dutch version of the Actionable questionnaire

The process of translating the English Actionable questionnaire into the Dutch language included forward translations and back-translations. The forward translator was a health professional (PJJ), familiar with the terminology of the area covered by the questionnaire. He is knowledgeable of the English-speaking culture but his mother tongue is Dutch. The translation was conceptual rather than literal, and natural and acceptable language for the broadest audience was used. Using the same approach as outlined above, the questionnaire was then translated back into English by an independent translator (TH), whose mother tongue is English and who has no knowledge of the questionnaire. As in the initial translation, emphasis in the back-translation was on conceptual and cultural equivalence and not linguistic equivalence. This procedure was iterated twice until a satisfactory version was reached. Figure 1 shows the Dutch version of the Actionable questionnaire.

### Study population

**Fig. 2:**
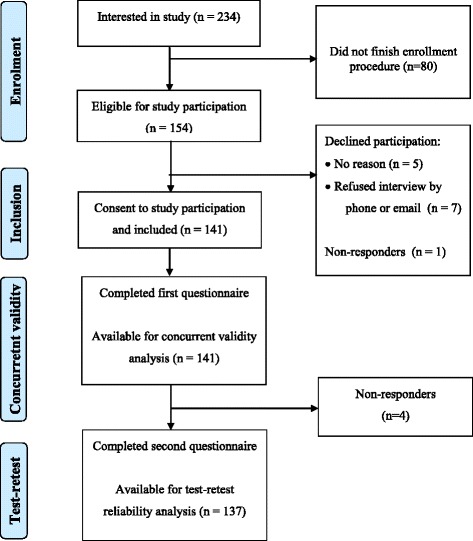
Numbers of patients involved in the enrolment phase, inclusion phase, concurrent validity analysis, and test-retest reliability analysis

Figure 2 shows the numbers of patients involved in the enrolment phase, inclusion phase, concurrent validity analysis, and test-retest reliability analysis. Of the 141 patients that were included, 106 were females and 35 males (female-to-male ratio 3.02). The age (mean, standard deviation [SD]) was 47.7 (10.40) years, the youngest participant being 24 years and the oldest 73 years. The disease course was relapsing remitting in 80, progressive in 48 and unknown in 13 patients. The Day 8 assessment was completed by 137 patients.

### Actionable, MSQoL-54, EDSS and MSIP values

The mean, SD, minimum and maximum values for the Actionable score, the Physical and Mental MSQoL-54 scores, the EDSS score and the MSIP domain and symptom disability scores are presented in Table 1.

**Table 1:** Actionable scores at test (Day 1) and retest (Day 8), and physical and mental MSQoL-54, EDSS, and MSIP domain and symptom disability scores

	Number	Mean	SD	Minimum	Maximum
Actionable score Day 1	141	8.00	3.94	0	18
Actionable score Day 8	137	7.61	4.00	0	21
MSQoL-54 Physical	138	53.32	17.42	15.00	96.60
MSQoL-54 Mental	138	64.88	16.92	21.15	95.40
EDSS	141	4.30	1.78	0.00	7.50
Muscle and Movement Functions	141	5.18	3.62	0.00	16.25
Excretion and Reproductive Functions	141	3.27	2.41	0.00	11.00
Mental Functions	141	2.65	1.93	0.00	9.00
Basic Movement Activities	141	3.38	3.60	0.00	15.00
Activities of Daily Living	141	6.82	6.06	0.00	24.00
Participation in Life Situations	141	4.30	4.53	0.00	18.00
Environmental Factors	141	4.78	3.87	0.00	16.00
Fatigue	136	0.38	0.60	0.00	3.00
Pain	141	2.06	0.96	0.00	4.00
Speech	141	0.94	0.85	0.00	4.00
Vision	131	0.72	0.86	0.00	4.00

*SD* standard deviation

### Test-retest reliability

Pearson’s r for the correlation between the Actionable scores at Day 1 and Day 8 was 0.8477 (*P* < 0.0001).

### Concurrent validity

Pearson’s coefficients for the correlations between the Actionable score at Day 1 and the scores for EDSS, MSQoL-54 Physical, MSQoL-54 Mental, and the MSIP domain and symptom disability scores are presented in Table 2. Statistically significant correlations were found between the Actionable Day 1 score and the EDSS (0.413, *P* < 0.0001), MSQoL-54 Physical (−0.308, *P* = 0.0002), MSQoL-54 Mental (−0.292, *P* = 0.0005), MSIP Muscle and Movement Functions (0.393, *P* < .0001), Excretion and Reproductive Functions (0.437, *P* < 0.0001), Mental Functions (0.196, *P* = 0.0189), Basic Movement Activities (0.373, *P* < 0.0001), Activities of Daily Living (0.322, *P* < 0.0001), Participation in Life Situations (0.286, *P* = 0.0006), Fatigue (0.184, *P* = 0.032), and Speech (0.171, *P* = 0.0426).

**Table 2:** Pearson’s coefficients for the correlations between the Day 1 actionable score and the EDSS, the MSQoL-54 Physical and Mental, and the MSIP domains and symptom disability scores

	Number	Pearson’s r	*P*
EDSS	141	0.413	<0.0001
MSQoL-54 Physical	138	−0.308	0.0002
MSQoL-54 Mental	138	−0.292	0.0005
Muscle and Movement Functions	141	0.393	<0.0001
Excretion and Reproductive Functions	141	0.437	<0.0001
Mental Functions	141	0.196	0.0189
Basic Movement Activities	141	0.373	<0.0001
Activities of Daily Living	141	0.322	<0.0001
Participation in Life Situations	141	0.286	0.0006
Environmental Factors	141	0.013	0.8775
Fatigue	136	0.184	0.0320
Pain	141	0.104	0.2197
Speech	141	0.171	0.0426
Vision	131	0.101	0.2130

### Item 9

At Day 1, 57 (40.42 %) patients answered that they would like to receive help for their bladder symptoms, whereas 84 (59.57 %) would not like to receive help. At Day 8, these figures were 61 (44.53 %) and 76 (55.47 %) respectively, yielding an r value for the correlation between Day 1 and Day 8 of 0.749 (*P* < 0.0001). Patients who would like to receive help (Day 1) had higher Actionable scores (10.32 [3.84] vs. 6.43 [3.18], *P* < 0.0001) (Day 1), lower physical MSQoL-54 scores (49.25 [16.89] vs. 56.01 [17.33], *P* = 0.0244), higher EDSS scores (4.74 [1.69] vs. 4.00 [1.78], *P* = 0.0131), and higher MSIP disability scores regarding Muscle and Movement Functions (6.14 [3.69] vs. 4.53 [3.44], *P* = 0.0089), Excretion and Reproductive Functions (4.39 [2.37] vs. 2.51 [2.13], *P* < 0.0001), Mental Functions (3.07 [2.01] vs. 2.36 [1.82], *P* = 0.0318), Basic Movement Activities (4.54 vs. 2.58 [3.16], *P* = 0.0013), Activities of Daily Living (8.18 [6.53] vs. 5.89 [5.57], *P* = 0.0276), Participation in Life Situations (5.43 [5.12] vs. 3.53 [3.94], *P* = 0.0137), and fatigue (0.53 [0.71] vs. 0.28 [0.48], *P* = 0.0163).

## Discussion

In an online observational study in 141 patients we assessed the test-retest reliability and the concurrent validity of a Dutch version of the Actionable questionnaire, an 8-item screening tool for neurogenic bladder overactivity in MS. The age of our study population (mean [SD] 47.7 (10.40), minimum 24 and maximum 73 years) was quite similar to that of the population (*N* = 151) in the validation study of the English version (mean [SD] 48.2 (12.11), minimum 22 and maximum of 80 years) [7]. The Pearson coefficient for the correlation between the test and retest Actionable scores, that we obtained with one week interval, was 0.85, which indicates a good test-retest reliability. The concurrent validity with respect to MS-related disability was also good, given the correlation between the Actionable score and the EDSS (0.41), and correlations between the Actionable score and the MSIP disability scores for the domains Muscle and Movement Functions (0.40), Excretion and Reproductive Functions (0.44), Basic Movement Activities (0.37), Activities of Daily Living (0.32), and Participation in Life Situations (0.29). The correlations with disability relating to Mental Functions, Fatigue and Speech were less than 0.20. No correlations were found between the Actionable score and the domain Environmental Factors, and the symptoms Pain and Vision. In fact, the disabilities showing lower or no correlations, were those that either regarded specific symptoms (Fatigue, Speech, Pain, Vision) or non-physical domains (Mental Functions, Environmental Factors). Thus, overall the findings on the Actionable’s concurrent validity with respect to MS-related disabilities were as might be expected.

HRQoL is an overall measure of well-being from a patient’s perspective. Given the potential impact of neurogenic bladder symptoms on psychological well-being and social functioning, we expected significant correlations between the Actionable score and the MSQoL-54 composites. Indeed, the associations for the physical and mental MSQoL-54 composites were −0.31 and −0.29, respectively, and statistically highly significant, and thus underlined the concurrent validity of the Dutch Actionable questionnaire. In the original ABSST and Actionable validation studies the Overactive Bladder Questionnaire Short Form (OABq-SF) was used, consisting of a 6-item symptom-bother scale and a 13-item HRQoL scale. A Dutch version of the OABq-SF, however, was not available [14]. Therefore we chose the MSQoL-54, a psychometrically validated scale that is widely used to measure HRQoL in MS patients [15]. It is of note that the MSQOL-54 is limited in its coverage of visual function and bladder and bowel problems [15], which may explain that the correlations between Actionable and MSQoL-54 scores were somewhat lower than e.g. the correlation between the Actionable and EDSS.

A limitation of the study is that we did not ask the clinicians whether they would refer the patient to a urologist, so we could not compare the Actionable outcome with the clinical indication. It should also be noted that the Actionable questionnaire is a screening tool and not designed for diagnostic purposes [7]. It aims to select those MS patient who are likely to benefit from referral to an urologist, in terms of accurate diagnosis and bladder-specific treatment [7]. To achieve this goal the questionnaire may be used by neurologists, MS nurses, and general physicians in out-patient settings on a regular basis, both as a paper-and-pencil test, computer-based or web-based. In view of the growing possibilities for MS patients to test and screen themselves online, the Actionable may contribute to patient self-management as well. E.g. when bladder symptoms occur, worsen or change the questionnaire may constitute an online tool for self-screening. However, the use should be embedded in the multidisciplinary care processes as practiced in the patient’s out-patient clinic, and according to predetermined rules and agreements. It may be conceived that a positive self-test is automatically followed by a set of measures, like testing for a urinary infection and a more comprehensive assessment of bladder symptoms and function.

Importantly, the optimal frequency of testing has not been established, neither in general nor in specific settings. A frequent usage could lead to an increased risk of false-positive outcomes. Moreover, the practicability of the test in real life practice, its clinical relevance (in terms of extra urological referrals, more urological diagnoses and effective bladder-specific treatments) and cost-effectiveness have not been investigated. The test’s limited number of items and the established high accuracy of the original English version [7] suggest that the implementation in daily practice may not be major problem and that the percentage of unnecessary urological referrals will be limited. Yet, future studies should address these questions.

## Conclusions

In a web-based observational study we validated a Dutch version of the 8-item Actionable screening questionnaire for neurogenic bladder overactivity in MS patients. The Dutch Actionable showed a good test-retest reliability and a good concurrent validity with disabilities and HRQoL.
